# Estimating district HIV prevalence in Zambia using small-area estimation methods (SAE)

**DOI:** 10.1186/s12963-022-00286-3

**Published:** 2022-02-19

**Authors:** Chris Mweemba, Peter Hangoma, Isaac Fwemba, Wilbroad Mutale, Felix Masiye

**Affiliations:** 1 Department of Health Policy, Systems and Management, School of Public Health, University of Zambia, Ridgeway Campus, P.O. Box 50110, Lusaka, Zambia; 2 School of Public Health, University of Ghana, P.O. Box LG 571, Accra, Ghana; 3 Department of Economics, School of Humanities and Social Science, University of Zambia, Great East Road Campus, P.O Box 32379, Lusaka, Zambia

**Keywords:** SAE, Small-area estimation, HIV, Prevalence, District, Fay–Herriot, Auxiliary information

## Abstract

**Background:**

The HIV/AIDS pandemic has had a very devastating impact at a global level, with the Eastern and Southern African region being the hardest hit. The considerable geographical variation in the pandemic means varying impact of the disease in different settings, requiring differentiated interventions. While information on the prevalence of HIV at regional and national levels is readily available, the burden of the disease at smaller area levels, where health services are organized and delivered, is not well documented. This affects the targeting of HIV resources. There is need, therefore, for studies to estimate HIV prevalence at appropriate levels to improve HIV-related planning and resource allocation.

**Methods:**

We estimated the district-level prevalence of HIV using Small-Area Estimation (SAE) technique by utilizing the 2016 Zambia Population-Based HIV Impact Assessment Survey (ZAMPHIA) data and auxiliary data from the 2010 Zambian Census of Population and Housing and the HIV sentinel surveillance data from selected antenatal care clinics (ANC). SAE models were fitted in R Programming to ascertain the best HIV predicting model. We then used the Fay–Herriot (FH) model to obtain weighted, more precise and reliable HIV prevalence for all the districts.

**Results:**

The results revealed variations in the district HIV prevalence in Zambia, with the prevalence ranging from as low as 4.2% to as high as 23.5%. Approximately 32% of the districts (*n* = 24) had HIV prevalence above the national average, with one district having almost twice as much prevalence as the national level. Some rural districts have very high HIV prevalence rates.

**Conclusions:**

HIV prevalence in Zambian is highest in districts located near international borders, along the main transit routes and adjacent to other districts with very high prevalence. The variations in the burden of HIV across districts in Zambia point to the need for a differentiated approach in HIV programming within the country. HIV resources need to be prioritized toward districts with high population mobility.

**Supplementary Information:**

The online version contains supplementary material available at 10.1186/s12963-022-00286-3.

## Background

The HIV/AIDS pandemic has continued to be a global public health problem, with an estimated 38 million people globally living with HIV in 2019 and the African region bearing the largest burden of the global HIV/AIDS cases [1]. Interestingly, the burden of HIV varies considerably within Africa, with sub-Saharan Africa alone accounting for about 70% of all global HIV cases in SSA [2]. However, a closer review of HIV in the SSA region reveals that the burden is mainly in Eastern and Southern African region (ESA) where, with only 6.2% of the world population, the ESA region accounted for approximately 54% of the total global HIV infections and 43% of all AIDS-related deaths in 2019 [1]. There is substantial variation in the distribution of HIV within the ESA region. For instance, of the 24 countries in this region, more than a quarter of the new HIV infections in 2018 were in South Africa, while 50% of infections were in 7 other countries, namely, and in order of magnitude, Mozambique, Tanzania, Uganda, Zambia, Kenya, Malawi and Zimbabwe [3].

Similarly, the distribution of HIV within countries has been shown to vary remarkably. In Zambia, for instance, some provinces such as Lusaka (16.1%), Western (16%) and Copperbelt (14.2%) have relatively high prevalence compared to provinces like North-western (6.9%) and Muchinga (5.9%) (ZAMPHIA, 2016). This trend is similar for South Africa where the burden of HIV among adult South Africans in 2016 ranged from as low as 12.6% in Western Cape to as high as 27% in Kwazulu-Natal (KZN) [4].

The information on the geographical variation in HIV prevalence at provincial level is certainly important for guiding government policy, prioritization of interventions and resource allocation both across and within countries. It should, however, be noted that the burden of diseases within the provinces can be heterogeneous. For example, within KZN province in South Africa, the available district-specific HIV prevalence in 2016 ranged from 16.1% in ILembe to 20.6% in uMgungundlovu [5]. Similarly, a study that modeled district-level estimates for HIV prevalence in South Africa found variations in the prevalence within the South African provinces [6]. This means that effective preventive and control strategies to combat HIV require knowledge of the burden of the disease at smaller and more similar areas such as districts [7]. This is challenging, however, because most data currently in use are not sufficiently powered to provide reliable estimates at the small-area levels such as districts [7].

The Zambian Ministry of Health acknowledges the importance of district-level estimates for more focused approaches in HIV programming and in facilitating the achievement of the Fast Track targets [8]. These targets are a set of 10 global guidelines for countries to adopt and implement in order to end the HIV pandemic by 2030 through ensuring, among other things, zero new HIV infections, zero discrimination and zero AIDS-related deaths. The Zambian MoH also acknowledges the importance of district-level HIV estimates in the achievement of these targets in an equitable manner. However, information on the prevalence of HIV at the district level is very limited and the MoH’s Fast Track strategies are unlikely to be realized. Currently, existing districts estimates for HIV in Zambia are from routine health facility data which cannot be generalized to the general population due to the non-random nature of the people that present to test for HIV [9]. This problem can only be remedied with the use of robust techniques, such as SAE methods, designed to provide valid estimates of the burden of HIV at district level.

District-level HIV statistics are of particular importance for Zambia because, a district is the lowest level of decentralization where health services are organized and delivered [10]. A previous study by Dwyer-Lindgren et al. [11] produced HIV prevalence estimates at a 5 × 5 km pixel resolution for countries in sub-Saharan Africa, including Zambia, which can be aggregated to the district level. However, the Dwyer-Lindgren et al. model is very computationally intensive, and not specifically tailored for Zambia. Our study, on the other hand, uses novel methodology that are specifically tailored for Zambia and can easily be replicated in other country-specific contexts.

## Methods

The district HIV prevalence was estimated using Small-Area Estimation (SAE) methods by utilizing multiple data sources. The SAE method is a statistical technique for obtaining reliable statistics for small areas that are mostly underrepresented in existing data sources due to small sample sizes. Using both direct and indirect methods, SAE models combine multiple data sources (censuses, surveys, etc.) containing other related information—auxiliary data—for these small areas [12].

Put simply, small-area estimates for HIV prevalence are a weighted average of the direct prevalence estimate from existing data which, due to sample size, may be too unreliable, and therefore requiring a statistical model that utilizes auxiliary data from outside the survey to improve the estimates [13]. More weight is placed on the predicted prevalence if the variance of the direct prevalence is high, and vice versa [6].

### Data sources

The outcome variable was HIV prevalence—obtained from the ZAMPHIA of 2016, while auxiliary predictors included HIV prevalence among pregnant women, the 2010 Zambian population; proportion of the population aged 15–36 years; dependence ratio (the ratio of population aged 0–14 years and persons aged 65 years and older per 100 persons in the working age group 15–64 years old [14]); the proportion of the population in formal dwelling; proportion of the population with higher education attainment; proportion of the population residing in the urban area, population density and the proportion of females in the population. Data on HIV prevalence among pregnant women were obtained from selected ANC facilities in the 74 districts in 2017 and 2018, while the rest of the auxiliary predictors were obtained from the 2010 Census of Population and Housing for Zambia.

The ZAMPHIA is a nationally representative cross-sectional, population-based survey of households across Zambia, aimed at measuring the status of Zambia’s national HIV response [15]. The 2016 ZAMPHIA used a two-stage stratified cluster sampling. The first stage selected 511 enumeration areas (EAs) using probability proportional to size method, and the second stage selected an average of 27 households per EA using equal probability method. A total of 13,441 households and 28,142 individuals were sampled for the survey; 19,168 were adults aged 15–59 years, and 8974 were children aged 0 to 14 years. Those aged 15–59 years received home-based counselling and testing for HIV. Additional information on the ZAMPHIA methodology is provided in the ZAMPHIA report [15].

Our study and the Dwyer-Lindgren study, alluded to earlier, have similar data sources, with both studies having utilized the ZAMPHIA and the ANC sentinel surveillance data, for HIV seroprevalence estimates and HIV prevalence among pregnant women, respectively. However, in addition to the above sources, the Dwyer-Lindgren study also uses the Zambia Demographic and Health Survey (ZDHS) for HIV seroprevalence estimates, while our study uses the Zambia 2010 Census of Population and Housing to obtain additional auxiliary predictors for HIV. Both studies offer very useful insights for better targeting of HIV resources at district level and facilitating the achievement of the Ministry of Health’s strategic HIV goals.

### Variable description

HIV prevalence is the number of HIV positive cases per 100 people tested for HIV in the ZAMPHIA and in the selected ANC clinics dotted across all the district. According to the 2010 Census of Population and Housing [14], population density is the total number of persons per square kilometer; proportion of urban area is the area considered to be urban out of the total area of the district; formal dwelling is defined as a room/set of rooms in a permanent building that could be structurally separated from a permanent building; dependence ratio is the ratio of the economically inactive persons to a 100 economically active persons; and higher education is the proportion of the population that have attained tertiary education.

### Statistical models

This study used the SAE technique to model and estimate HIV prevalence in Zambia, adapting methods from a similar study in South Africa [6]. Note that the outcome variable entered the modeling framework as a logit transformation of the direct district HIV prevalence from the ZAMPHIA survey. The ANC HIV prevalence rate was also modeled as a logit transformation. The HIV prevalence rates are the direct domain estimates of the Zambian district-level HIV prevalence proportions from the ZAMPHIA survey, while the ANC HIV prevalence rates are the prevalence proportions among pregnant women who obtained antenatal care services from clinics dotted across the various districts in Zambia. The logit transformation was necessary for converting prevalence proportions to the real line which helps in ascertaining the normality assumption test. Similarly, sampling error variance was estimated as Delta-method approximation using the variances of the domain estimates as reported and elaborated elsewhere [5]. The model estimated the true HIV prevalence by combining the direct estimate (i.e., direct methods estimation) from the ZAMPHIA survey and the indirect model-based estimates, based on auxiliary predictors and the spatial correlation effects meant to improve the model prediction by borrowing strength from across the districts [6]. The direct estimate of HIV prevalence, $\overline{y}_{i}$ for district i, was obtained as a weighted mean district-specific HIV prevalence from the ZAMPHIA survey. This estimate can be viewed to be as follows:

1 $$\overline{y}_{i} = \Theta_{i} + \varepsilon_{i}$$

where $\overline{y}_{i}$ is the HIV prevalence estimate for district *i* estimated from the survey data; $\Theta_{i}$ is the district’s true HIV prevalence being estimated; and *ε*_*i*_ is the random error with mean 0 and variance $\sigma_{i}^{2}$ and is assumed to be normally distributed.

However, since the number of respondents sampled at district level, during the ZAMPHIA, is not sufficient to provide reliable district HIV prevalence estimates, the second part of the model, referred to as indirect method, was estimated to improve the reliability of the estimates. Therefore, in addition to the direct prevalence estimates obtained from ZAMPHIA, the indirect method used auxiliary information from within the district and neighboring districts, and other data sources to borrow strength and improve the precision of the HIV prevalence estimates [16]. Since the outcome variable was a logit transformation of HIV prevalence, we assumed that HIV prevalence is a linear function of covariates or HIV risk factors obtained from auxiliary data [6]. The true HIV prevalence ($\Theta_{i}$ in Eq. 1) can therefore be thought of as:

2 $$\Theta_{i} = x_{i} \beta + v_{i}$$

*β* is a set of regression coefficients obtained by regressing $\overline{y}_{i}$ on HIV risk factors (*x*_*s*_) and *v*_*i*_ are normally distributed random errors with mean 0 and variance $\sigma_{v}^{2}$. Note that $\sigma_{v}^{2}$ and $\sigma_{i}^{2}$ are independent of each other. Combining Eqs. 1 and 2 gives the following mixed-effects linear regression model;

3 $$\overline{y}_{i} = x_{i} \beta + v_{i} + \varepsilon_{i}$$

To improve precision of the HIV prevalence estimates from Eq. 3, there is need for a model that combines direct and indirect estimates into a single estimate, such as the Fay–Herriot (FH) small-area estimator. The FH estimator is a linear combination of a direct and synthetic estimator which reduces estimation variance in the underrepresented small areas and in the whole model [17]. The FH estimator is given by:

4 $$\overline{y}_{i} = \gamma_{i} \overline{y}_{i} + \left( {1 - \gamma_{i} } \right)x_{i} \hat{\beta }$$

where *γ*_*i*_ and 1 − *γ*_*i*_ are weights for the direct estimate $\overline{y}_{i}$ and the synthetic estimate, $x_{i} \hat{\beta }$, respectively, which constitute the FH estimator. Note that *γ* is simply the ratio of the model error variance to the total error, i.e., $\frac{{\sigma_{v}^{2} }}{{\sigma_{v}^{2} + \sigma_{i}^{2} }}$. This means that if the survey-based estimates are precise, more weight is given to the direct estimate. Similarly, low precision of the survey-based estimates results in more weight being given to the synthetic or indirect estimate.

### Spatial correlation

There is evidence that areas close to each other tend to have similar population dynamics, such as disease risk factors and disease burden [18]. This highlights the importance of location and geographical clustering in determining the spread of, and burden of disease—especially infectious diseases, for areas that are in close proximity [19, 20]. A study in Ethiopia documented the importance of geographical clustering in determining the prevalence of HIV and Tuberculosis (TB) [21].

To account for this spatial correlation, we built a spatial Fay–Herriot (SFH) model and tested it against a non-spatial model to ascertain the best fitting model for this study. A spatial adjacency matrix (*W*) was built in Excel, as follows:

Spatial adjacency matrix (*W*) is an *n* × n matrix where n is the number of district in Zambia.

The diagonal entries are *W*_*ii*_ = 0, indicating no correlation for district *i* to itself.

The off-diagonal row entries add up to 1, i.e., *W*_*ij*_ = 1. This can be thought of as follows, as presented by Yakoi and Ando [22];

5 $$w_{1ij} = \left\{ {\begin{array}{*{20}c} {1/d_{ij, }^{\alpha } } \\ 0 \\ \end{array} } \right.$$

$${\mathrm{If}}\,i \ne j\,{\mathrm{otherwise}}$$

6 $$w_{oij} = w_{ij} /\mathop \sum \limits_{k = 1}^{N} w_{1ik}$$

where *d*_*ij*_, in Eq. (5), is the distance between districts *i* and *j*; *α* is a parameter of the distance decay (*α* = 0 if *ij* do not share a border, otherwise 0 < *α* < 1). According to Eq. (6), the total amount of influence that one area receives from other areas is fixed [22].

The data analysis was conducted in R [23] utilizing the SAE package built in the software [24]. Figures were produced with the ggplot2 package [25].

### Model selection

We fitted a variation of basic area-level models which differed in the inclusion of auxiliary predictors and assumptions about the random effects. Model 1 included only the logit of ANC prevalence proportion as an auxiliary predictor. Models 2–9 augmented model 1 with inclusion of the district-level percentages of dependency ratio (DR), formal dwelling (Formal), high education (HE), land considered to be urban (Urban), district population (Pop), population aged between 15 and 35 years (15–35 years), population density (PD) and female population (Female), respectively.

Model 10 augmented model 2 with inclusion of formal dwelling. Model 11 augmented model 10 with inclusion of higher education. Model 12 augmented model 11 with inclusion of urban prop. Model 13 augmented model 12 with inclusion of pop2010. This continued until model 15, which augmented model 14 with the inclusion of pop density. Model 16 augmented model 15 with female population.

Model 17 was reduced from model 16 by deletion of the logit of ANC prevalence and provides the contrast needed to assess the value of ANC prevalence. Models 18–35 relaxed the assumption of independent model errors in models 1, 2 through to 17, respectively, with inclusion of a simultaneously autoregressive (SAR) spatial covariance structure. Model 35 only contained the SAR covariance structure without any covariates. The spatial adjacency matrix, described earlier, accounted for the SAR covariance structure. Relative model performance was assessed using the Akaike Information Criterion (AIC). The AIC balances model fit against model complexity; smaller values of AIC indicate relatively better predictive ability. AIC is a dimensionless relative measure, and according to Gutreuter and others [6], differences of 5 between models are customarily considered to be important.

District-level estimates of the burden of HIV infection were estimated from the best fitting model (Model 19) which included the logit of ANC prevalence proportion and dependence ratio with the SAR spatial covariance structure. This model was thereafter used, in combination with the survey-based HIV prevalence estimates, to model the prevalence of HIV in all the 74 districts of Zambia. A table containing information on the fitted models has been included as an appendix (See Additional file 1).

Note that there are other models that can be used to account for autocorrelation effect, such as the conditional autoregressive (CAR) model, and its intrinsic version (intrinsic autoregressive [IAR] model), and the decision to use SAR is because these models are equivalent and in practice produce similar results [26, 27].

Note that there are differences in the modeling approaches between our study and the comparable study by Dwyer-Lindgren and others [11]. Our study was based on small-area estimation process, while the Dwyer-Lindgren study focuses on estimating the sub-national variation of HIV prevalence using within-country variation at a 5 × 5-km resolution. Further, the paper reported use of a cross-walking model to link disparate data sources that leveraged existing microdata and linear regression estimates. Use of k-means clustering to generate a reduced set of locations based on the centroid of each k-means cluster helped to generate pseudo-points which were assigned to HIV prevalence observed for the polygon as a whole. This is different from our paper, where district-level data were obtained and not estimated or assumed. All the estimates in our study were linked to available survey data which helped to provide associated survey parameters.

Further, Dwyer-Lindgren et al. fitted three sub-models to the HIV survey data using generalized additive models, boosted regression trees and lasso regression. They implemented geostatistical modeling framework which allowed them to model HIV prevalence using a spatially and temporally explicit generalized linear mixed effects model. Unlike in our model, their logit-transformed HIV prevalence was modeled as a linear combination of a regional intercept, covariate effects, country random effects, spatially and temporally correlated random effects. In our modeling framework, temporality seasonal effect was not included even though the effect of the spatial term was done. Note also that the frequentist approach was the main inference strategy for our study, while Dwyer-Lindgren et al. used Bayesian framework with a deterministic approach. Their model used the stochastic partial differential equation approach to approximate the continuous spatial and spatiotemporal Gaussian random fields. We note that this was appropriate given the complexity of their dataset which would have suffered from serious computation cost if the frequentist or the sampling-based approach was implemented.

## Results

Table 1 shows the population demographics of the auxiliary predictors used to predict district HIV prevalence. For instance, it can be seen that the population aged 15–35 years represented about 35% of the population, although it ranged from the lowest rate of about 32% in some districts to highest of almost 45% in other district. The median population with higher education was 3.3% (ranged from 1.2 to 16%), while the median population of HIV positive pregnant women was approximately 26%. The females made up of 50.8% of the population. Table 1 provides more details.

**Table 1:** Population demographics of the auxiliary predictors

Auxiliary predictors	Mean (median)	Min.–Max
Population aged 15–35 years	35.2 (34.4)	31.7–44.9
Dependence ratio	99.8 (103.1)	64.7–114.9
Population living in formal dwelling	15.8 (8.3)	0.76–88.8
Population with higher Education	4.6 (3.3)	1.2–16.1
Urban area	25.4 (14.7)	0–100
HIV among pregnant women at ANC clinics	31.6 (25.5)	2–90
Population density	107.9 (15.8)	2.8–4853
Female population	50.8 (50.8)	49.2–53.4

### Model diagnosis and validation

**Fig. 1:**
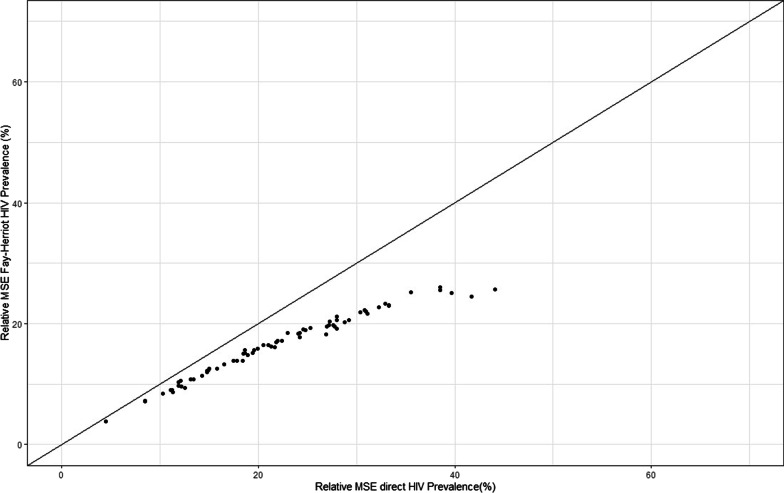
Relative mean standard errors (RMSE) for the FH HIV prevalence estimates and survey-based prevalence estimates: The RMSE show lower mean standard errors for the Fay–Herriot small-area estimations over the survey-based estimation for all the 74 Zambian districts

The results obtained using the SAE estimates model were consistently more precise than those obtained from the direct estimate methodology. For instance, the relative mean standard errors (RMSE) in Fig. 1 and the relative standard errors (See Additional file 2) for the SAE are continuously lower than those from the direct estimate model. In addition, the reduction in relative standard errors, due to SAE, was greatest in districts which produced the least precise direct estimates. For instance, districts like Chadiza, Milenge, Gwembe and Chavuma have relative standard errors reducing from 99.7 to 30.7%, 70.2 to 30%, 70.9 to 29.5% and 70.4 to 33.1%, respectively. Assuming, for example, that “useful” estimates are those for which RSE ≤ 20%, then our SAE model produced useful estimates in 52 of the 74 districts for which direct estimation failed to produce useful estimates.

**Fig. 2:**
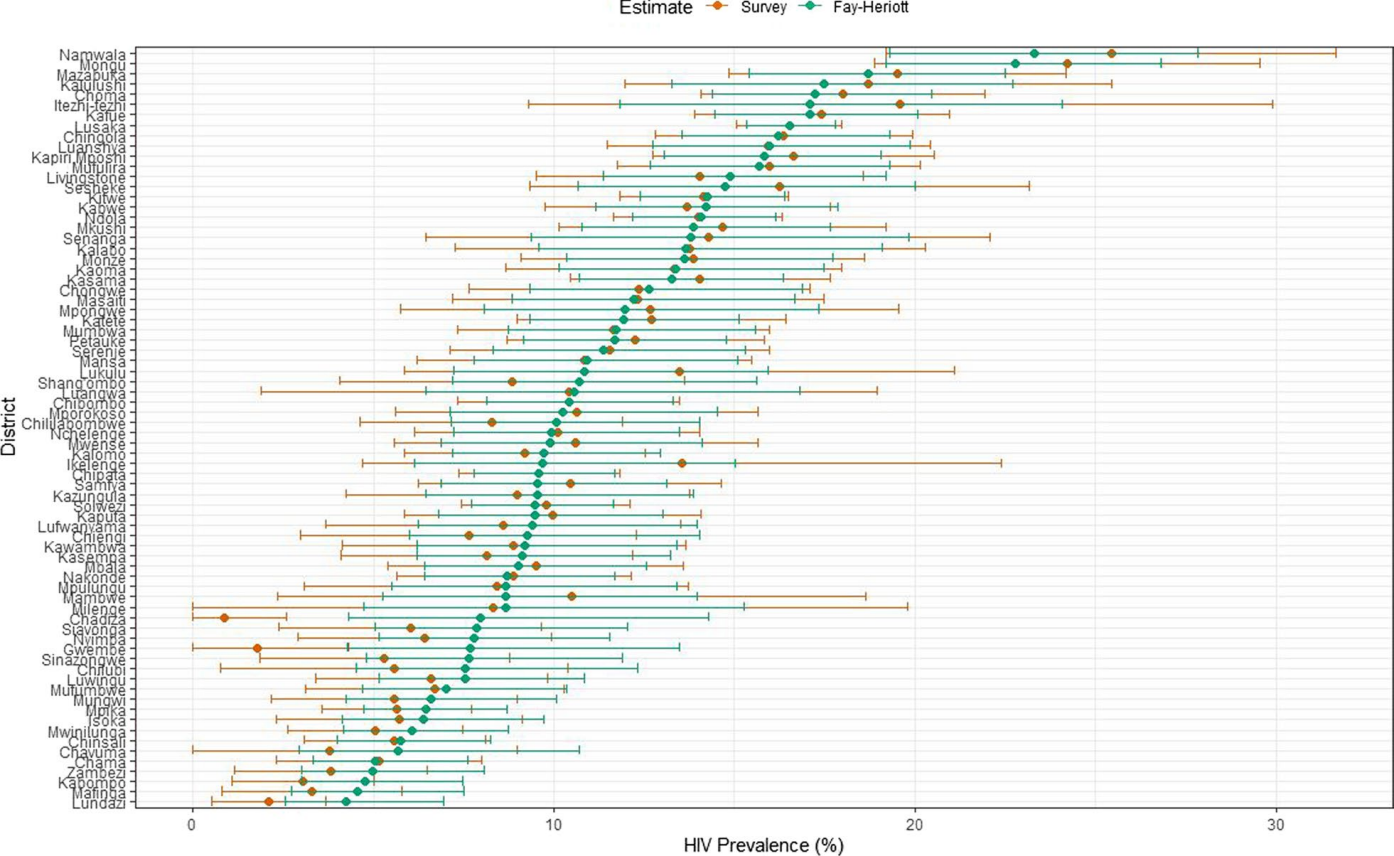
HIV prevalence estimates and confidence intervals for the FH and direct estimates in Zambia’s districts: The confidence intervals of the FH estimates are narrower than those of the direct estimates for most of the districts

It is worth noting that the estimates from the Fay–Herriot estimator had narrower 95% confidence intervals than the direct estimates (See Fig. 2). Conversely, some point estimates for some districts such as Chadiza and Gwembe differed rather substantially between the design-based and model-based estimates. The design-based survey domain estimate of HIV prevalence in Gwembe and Chadiza was of little value for lack of precision, and at most misleading. Smaller relative standard errors from the FH small-area estimates are more likely to be true, compared to those from the direct estimates, and are much more likely to be similar to surrounding districts.

The conclusion from this model diagnostics and validation is that the FH estimator produces smaller standard errors compared to the survey-based estimates, across all the 74 districts of Zambia. This means that SAE prevalence estimates are more reliable than those obtained from the direct estimates.

### District HIV prevalence estimates

The district HIV prevalence in Zambia ranges from as low as 4.3% (CI 2.6–6.9) in Lundazi to as high as 23.3% (CI 19.3–27.8) in Namwala. Other notable districts with high HIV prevalence, in order of magnitude, include Mongu (22.8%; CI 19.2–26.8), Mazabuka (18.7%; 15.4–22.5), Kalulushi (17.5%; CI 13.2–22.7), Choma (17.2%; CI 14.4–20.5), Itezhi-tezhi (17.1%; CI 11.8–24.1), Kafue (17.1%; CI 14.4–20.1) and Lusaka (16.5%; CI 15.3–17.8). On the other hand, the five districts with the lowest HIV prevalence, in descending order, were: Chama (5%; 3.3–7.6), Zambezi (4.9%; CI 3–8.1), Kabompo (4.8%; CI 2.9–7.5), Mafinga (4.6%; CI 2.7–7.5) and Lundazi (4.3%; CI 2.6–6.9). The results of the SAE reveal that 37 of the 74 districts had relatively low HIV prevalence (≤ 10%), 25 districts had relatively moderate HIV prevalence (between 10 and 15%), 10 districts had relatively high HIV prevalence (between 15 and 20%), while 2 districts had relatively very high HIV prevalence (between 18.1% and 23.5%). Table 2 (See Additional file 3) provides both direct and modeled HIV estimates for all the 74 districts, with confidence intervals.

**Fig. 3:**
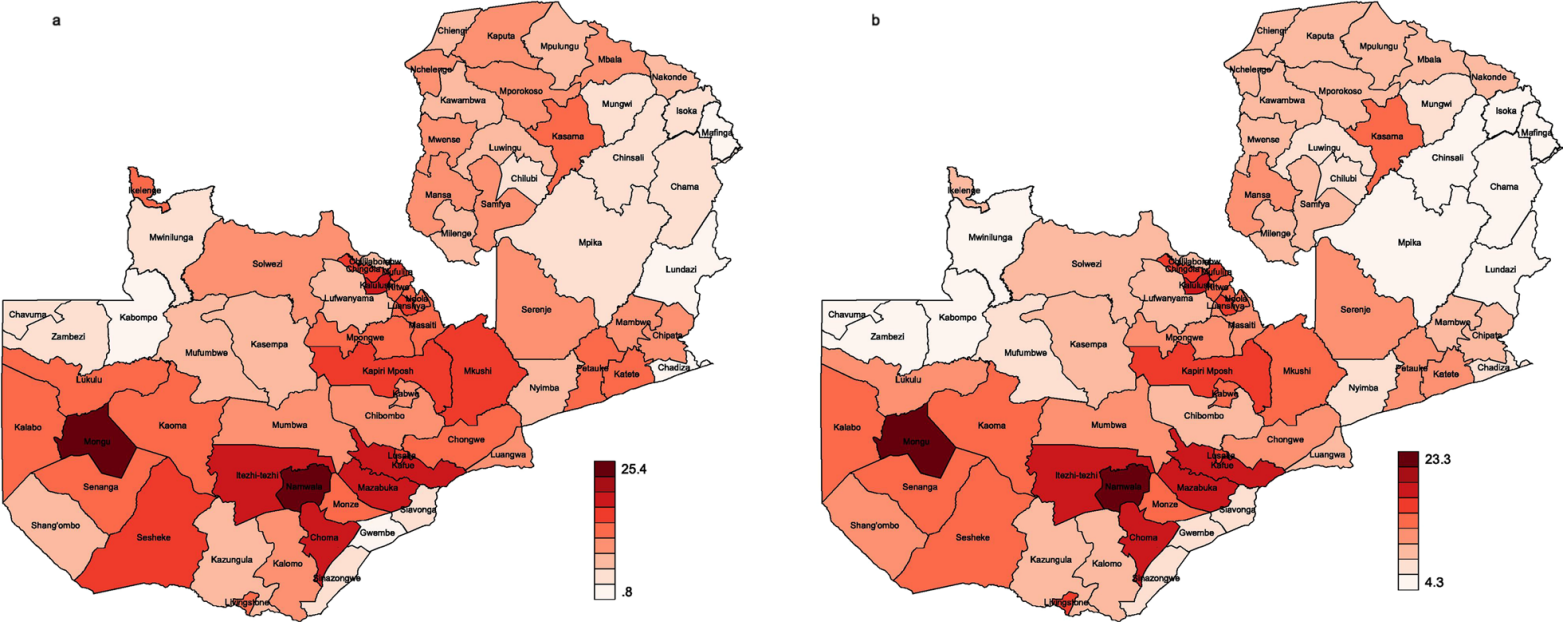
Zambia district HIV prevalence maps for raw (**a**) and SAE (**b**) data: The color variations in the heat map show the magnitude of the HIV prevalence in the 74 districts

The distribution of district HIV prevalence is further illustrated with the two maps in Fig. 3. Figure 3a shows the district prevalence map from the direct estimates, while Fig. 3b shows the map generated using SAE data. The notable difference between the maps is that the one developed using raw data has a wider HIV prevalence interval (0.8–25.4%) compared to the SAE map (4.3–23.3%). The spatial effect of HIV prevalence can also be seen from the SAE map (Fig. 3b), with relatively high HIV being concentrated in areas around central, southern and western Zambia. Note, however, that the prevalence intensities in maps 3a and 3b are based on relative prevalence between the lowest and highest prevalence estimates within each map. Therefore, comparing the two maps should be done with caution.

The mapping shows that, generally, the districts in the north and eastern parts of the country have moderate HIV prevalence, while districts in north-western and north eastern parts of the country, i.e., North-Western and Muchinga provinces, have the lowest HIV prevalence.

## Discussion

This paper is the first to use SAE methods to estimate the prevalence of HIV at district level in Zambia. Our study has demonstrated that national HIV estimates currently being used for HIV programming fail to account for the full picture of the distribution, and the extent of the variations in HIV prevalence at lower levels [6, 7, 28, 29]. Amoako Johnson [28], for instance, warns against relying on national estimates for planning as this could lead to an “ecological fallacy,” where planning and resource allocation fail to properly account for the variations that exist at small domains, but may not be apparent at national level. The one-size-fits-all approach, associated with national level estimates, is unlikely therefore to achieve the desired results at local levels [29].

In the midst of declining HIV funding [30], designing and targeting of HIV interventions require adequate knowledge on where the biggest resource needs lie. In the context of Zambia, for example, national HIV estimates would demand that more resources be allocated to the Western province, based on the disease burden. However, these national level estimates do not provide any information on the district-specific HIV burden, or sub-groups in greater need of HIV policy targeting within the province [31]. The revelations of the wide variations in the burden of HIV within districts should be a policy concern and effectively makes the “bigger picture” approaches redundant, especially if the intention is to make HIV programs more pragmatic and optimal at the local levels [32, 33]. The importance of accounting for within district variation in HIV prevalence has been highlighted by our study. For instance, while the average HIV prevalence for Southern province is around 13%, the within province prevalence varies from as low as 7.4% to as high as 23.5%. Ensuring effective service delivery, under such circumstances, requires recognizing and tailoring interventions to the needs of the different subpopulations at the level at which service delivery is organized and delivered [34]. This remains a challenge for low resource countries, however, due to the higher cost of obtaining data to generate small-area estimates [35].

Our study has also revealed important information on the predictors of HIV prevalence at district level. For instance, our study has shown that ANC HIV prevalence and dependence ratio are the best out of survey predictor for district HIV prevalence. This is similar to the HIV prediction model in South Africa [6], except the one in our study included an SAR spatial covariance structure. Other studies [36] have also found HIV prevalence among pregnant women to be a good predictor of adult HIV prevalence. On the other hand, dependence ratio may be influencing HIV prevalence indirectly, i.e., high dependence ratio negatively affects economic well-being [37], which increases the vulnerability of the population and their susceptibility to HIV [38, 39].

Another important finding in this study is that district HIV prevalence in Zambia is spatially correlated, i.e., the prevalence in one district is correlated with the prevalence in adjacent districts. This is reasonable and expected since district boundaries are arbitrary, and therefore, individuals living in districts close to each other are likely to have similar characteristics and risk factors [28, 40]. Similar studies have acknowledged the importance of accounting for spatial correlation at small-area levels [6, 28], and this is especially true for communicable diseases such as HIV. It would be prudent, therefore, for neighboring districts to employ coordinated approaches to HIV programming and have a shared understanding of local HIV drivers and impact of the disease. The mapping of HIV prevalence in our study provides useful information to facilitate such a coordinated HIV response.

The national HIV prevalence for Zambia has generally been highest in urban areas [15, 41, 42], and this is similar to other countries in the region such as Malawi, Kenya, South Africa and Zimbabwe [43–46]. However, district-level estimates from our study have revealed that HIV prevalence in some rural districts is comparable, and sometimes even higher than the prevalence in urban districts. For instance, we found that the two highest HIV prevalence estimates in Zambia are in predominantly rural districts, with the highest district having almost seven percentage points higher prevalence than that of the most urbanized district of Lusaka. This is further proof that national-level estimates mask very important HIV dynamics that can guide resource allocation at local levels [47]. It is likely that the national-level HIV dynamics observed in most countries are different to the situation at lower levels. As long as lower-level prevalence estimates remain unknown, the allocation of HIV resources will remain sub-optimal [48].

The lessons that can be learnt from our study are that HIV prevalence is highest in districts located near international borders, along the main transit routes and adjacent to other districts with very high prevalence. Such districts tend to have high population mobility due to commerce and trade. Similarly, the two rural districts with the highest HIV prevalence in Zambia are fishing districts and attract a large number of people for fish rated trade every year [49–52]. Population mobility has been shown to be a driver of HIV infections in other settings as well [53–55]. Other similar countries can draw important lessons from this finding. To demonstrate the importance of population mobility in HIV transmission, our study found that districts that experience seasonality of employment located along the main transit routes and those along the international border have higher HIV prevalence than the national average. The above factors have been shown to be associated with HIV in other settings as well [56–58]. Districts experiencing high population mobility are potential HIV hotspots and should be prioritized for HIV interventions such as test and treat services, regardless of location. Similarly, areas that are in close proximity to districts with known high HIV prevalence need close attention due to the spatial nature of the HIV epidemic, as revealed by our study.

There are some notable differences between our district HIV estimates and those of Dwyer-Lindgren et al., [11] a comparable study. For instance, our HIV prevalence estimates, which are based on the 2016 ZAMPHIA survey, ranged from 4.2% in Lundazi to 23.3% in Namwala, while the prevalence estimates for Dwyer-Lindgren and others, over the same period, ranged from 4.4% in Isoka to 17.9% in Mongu. The differences in results may be attributed to the different modeling principles employed by the two studies, or the fact that the Dwyer-Lindgren estimates are based on the age group 15–49 years with data from 9 provinces and 72 districts, while our estimates are based on the age group 15–59 years with data from 10 provinces and 74 districts.

## Conclusion

This is the first study in Zambia to present and map HIV prevalence estimates at district level using SAE methods. It is clear from the results that national estimates mask the wide variation in HIV prevalence within the districts. Ensuring that HIV resources are allocated where they are needed require knowledge on the distribution of HIV at smaller, more homogeneous areas such as districts. This study has been able to provide this information and mapped the distribution of district HIV in Zambia.

The revelation that HIV prevalence is very high in some rural districts is an important finding for HIV programming. It is useful for policy makers to realize that relying on national level prevalence to plan interventions at district level may not be optimal because the HIV dynamics at district level are likely to be different. Utilizing results from SAE techniques for planning and resource allocation would ensure achievement of universal access to resources by underserved and underrepresented populations.

Our results have documented drivers and markers of high HIV prevalence at district level; information that can be used to plan prevention and treatment interventions. Population mobility is a key driver of HIV and should be an important consideration when designing HIV interventions. Profiling the burden of disease at appropriate levels is a key aspect in designing responsive HIV interventions, and SAE models will increasingly become important tools in guiding policy making and decision making, especially for low resource settings.

### Study limitations

The SAE model used in this study helped produce district HIV prevalence estimate; however, the use of relative mean standard errors and confidence intervals to validate the model has a potential bias. It should be noted that ZAMPHIA is not designed to collect representative data at district level, and by design therefore, SAE methods are always going to produce relatively better estimates, with smaller standard errors than ZAMPHIA estimates because they utilize additional data, in addition to the survey-based estimates. An additional validation method would have been useful. Additionally, the model was built with covariates as collected by the Census data and ZAMPHIA, and there is a chance that other HIV-related covariates, not collected by the Census and the ZAMPHIA, e.g., the prevalence of transactional sex, could have strengthened the model. The other limitation is that this study uses data from different time points, i.e., the 2016 ZAMPHIA, 2017–18 ANC and 2010 census data, which may affect the observed relationship between the outcome and the explanatory covariates. It is, however, unlikely that any demographic changes over the review period would significantly change our findings. Generally, this study has provided policy relevant information that can be utilized to improve targeting of HIV resources at local levels where interventions are planned and delivered.

## Supplementary Information

**Additional file 1**. Fitted models to estimate district HIV prevalence in Zambia.

**Additional file 2**. Relative Standard Errors for direct and modeled HIV estimates.

**Additional file 3**. Direct and modeled HIV estimates with confidence intervals.

## Data Availability

The datasets used and/or analyzed during the current study are available from the corresponding author on reasonable request.

## Abbreviations

HIV/AIDS: Human immunodeficiency virus/acquired immunodeficiency syndrome
SAE: Small-area estimation
ZAMPHIA: Zambia population-based HIV impact assessment survey
ANC: Antenatal care
FH: Fay–Herriot
ESA: Eastern and Southern African region
KZN: Kwazulu-Natal
MoH: Ministry of Health
EA: Enumeration area
TB: Tuberculosis
SAR: Simultaneously autoregressive
CAR: Conditional autoregressive
IAR: Intrinsic autoregressive
AIC: Akaike information criterion
RMSE: Relative mean standard errors
UNZABREC: University of Zambia Biomedical Research Ethics Committee
ZamStats: Zambia Statistics Agency
US: United States
NIH: National Institutes of Health
