# Supplementary material for: Estimating district HIV prevalence in Zambia using small-area estimation methods (SAE)
Source: Popul Health Metr. 2022 Feb 19;20:8. doi: 10.1186/s12963-022-00286-3 (PMC8858531; doi:10.1186/s12963-022-00286-3)
Supplement: Supplementary file 1 — Additional file 1. Fitted models to estimate district HIV prevalence in Zambia. [file 12963_2022_286_MOESM1_ESM.docx]

**Appendix 1: Fitted models to estimate district HIV prevalence in Zambia**

ANC Prev = HIV prevalence among pregnant women in selected ANC clinics; DR=Dependence Ratio; Formal=Prevalence of formal dwelling; HE=Prevalence with higher education attainment; Urban=Prevalence of area considered urban; Pop=District population; 15-35yrs=Prevalence of population aged 15-35years; PD=Population density; Female=Prevalence of females; SAR= Simultaneously Autoregressive and LL=Log-likelihood

| Model | Covariates | Cov. Structure | LL | AIC |
| --- | --- | --- | --- | --- |
| 1 | Logit (ANC Prev) | Independence | -57.87 | 121.75 |
| 2 | Logit (ANC Prev) + DR | Independence | -51.65 | 111.31 |
| 3 | Logit (ANC Prev) + Formal | Independence | -53.24 | 114.48 |
| 4 | Logit (ANC Prev) + HE | Independence | -53.00 | 114.01 |
| 5 | Logit (ANC Prev) + Urban | Independence | -53.95 | 115.89 |
| 6 | Logit (ANC Prev) + Pop | Independence | -55.42 | 118.85 |
| 7 | Logit (ANC Prev) + 15-35yrs | Independence | -52.57 | 113.13 |
| 8 | Logit (ANC Prev) + PD | Independence | -56.58 | 121.16 |
| 9 | Logit (ANC Prev) + Female | Independence | -57.86 | 123.73 |
| 10 | Logit (ANC Prev) + DR + Formal | Independence | -51.60 | 113.20 |
| 11 | Logit (ANC Prev) + DR + Formal+ HE | Independence | -51.60 | 115.20 |
| 12 | Logit (ANC Prev) + DR + Formal+ HE + Urban | Independence | -51.15 | 116.30 |
| 13 | Logit (ANC Prev) + DR + Formal+ HE + Urban + Pop | Independence | -50.53 | 117.07 |
| 14 | Logit (ANC Prev) + DR + Formal+ HE + Urban + Pop + 15-35yrs | Independence | -50.53 | 119.05 |
| 15 | Logit (ANC Prev) + DR + Formal+ HE + Urban + Pop + 15-35yrs + PD | Independence | -49.89 | 119.77 |
| 16 | Logit (ANC Prev) + DR + Formal+ HE + Urban + Pop + 15-35yrs + PD + Female | Independence | -49.33 | 120.65 |
| 17 | DR + Formal+ HE + Urban + Pop + 15-35yrs + PD + Female | SAR | -52.24 | 124.49 |
| 18 | Logit (ANC Prev) + SAR | SAR | -53.77 | 115.53 |
| 19 | Logit (ANC Prev) + DR + SAR | SAR | -48.32 | 106.64 |
| 20 | Logit (ANC Prev) + Formal + SAR | SAR | -49.44 | 108.89 |
| 21 | Logit (ANC Prev) + HE + SAR | SAR | -49.27 | 108.55 |
| 22 | Logit (ANC Prev) + Urban + SAR | SAR | -49.90 | 109.80 |
| 23 | Logit (ANC Prev) + Pop +SAR | SAR | -52.44 | 114.88 |
| 24 | Logit (ANC Prev) + 15-35yrs + SAR | SAR | -49.12 | 108.25 |
| 25 | Logit (ANC Prev) + PD + SAR | SAR | -53.23 | 116.46 |
| 26 | Logit (ANC Prev) + Female + SAR | SAR | -53.76 | 117.52 |
| 27 | Logit (ANC Prev) + DR + Formal + SAR | SAR | -48.31 | 108.63 |
| 28 | Logit (ANC Prev) + DR + Formal + HE + SAR | SAR | -48.31 | 110.62 |
| 29 | Logit (ANC Prev) + DR + Formal + HE + Urban + SAR | SAR | -48.16 | 112.33 |
| 30 | Logit (ANC Prev) + DR + Formal + HE + Urban + Pop + SAR | SAR | -48.02 | 114.04 |
| 31 | Logit (ANC Prev) + DR + Formal + HE + Urban + Pop + 15-35yrs + SAR | SAR | -48.00 | 116.01 |
| 32 | Logit (ANC Prev) + DR + Formal + HE + Urban + Pop + 15-35yrs + PD + SAR | SAR | -46.97 | 115.95 |
| 33 | Logit (ANC Prev) + DR + Formal + HE + Urban + Pop + 15-35yrs + PD + Female + SAR | SAR | -46.93 | 117.87 |
| 34 | DR + Formal + HE + Urban + Pop + 15-35yrs + PD + Female + SAR | SAR | -47.83 | 117.67 |
| 35 | SAR | SAR | -55.44 | 116.88 |
