# Supplementary material for: Estimating district HIV prevalence in Zambia using small-area estimation methods (SAE)
Source: Popul Health Metr. 2022 Feb 19;20:8. doi: 10.1186/s12963-022-00286-3 (PMC8858531; doi:10.1186/s12963-022-00286-3)
Supplement: Supplementary file 2 — Additional file 2. Relative Standard Errors for direct and modeled HIV estimates. [file 12963_2022_286_MOESM2_ESM.docx]

**Appendix 2: Relative Standard Errors for direct and modeled HIV estimates**

| **District** | **RSE_SAE** | **RSE_Diirect** |  | **District** | **RSE_SAE** | **RSE_Direct** |
| --- | --- | --- | --- | --- | --- | --- |
| Chibombo | 12.5 | 15 |  | Mafinga | 26 | 38.5 |
| Kabwe | 12 | 14.8 |  | Mpika | 15.5 | 18.6 |
| Kapiri Mposhi | 9.6 | 11.9 |  | Nakonde | 15.3 | 18.6 |
| Mkushi | 12.6 | 15.8 |  | Chilubi | 25.9 | 44.1 |
| Mumbwa | 14.8 | 18.9 |  | Kaputa | 16.5 | 21 |
| Serenje | 15.5 | 19.6 |  | Kasama | 10.8 | 13.1 |
| Chililabombwe | 17.3 | 22.4 |  | Luwingu | 18.9 | 24.8 |
| Chingola | 9 | 11.1 |  | Mbala | 17.2 | 22 |
| Kalulushi | 13.8 | 18.4 |  | Mporokoso | 18.1 | 24.1 |
| Kitwe | 7.1 | 8.5 |  | Mpulungu | 22.1 | 32.3 |
| Luanshya | 11.4 | 14.3 |  | Mungwi | 22.1 | 31 |
| Lufwanyama | 20.8 | 29.2 |  | Chavuma | 33.7 | 70.4 |
| Masaiti | 16.3 | 21.3 |  | Ikelenge | 23.2 | 33.3 |
| Mpongwe | 19.6 | 27.8 |  | Kabompo | 23.4 | 32.9 |
| Mufulira | 10.8 | 13.4 |  | Kasempa | 19.1 | 25.3 |
| Ndola | 7.2 | 8.5 |  | Mufumbwe | 20.2 | 27.3 |
| Chadiza | 31 | 99.7 |  | Mwinilunga | 19 | 24.6 |
| Chipata | 10.4 | 11.9 |  | Solwezi | 10.5 | 12.1 |
| Katete | 12.4 | 14.9 |  | Zambezi | 25.3 | 35.5 |
| Lundazi | 25.6 | 38.5 |  | Choma | 9 | 11.2 |
| Mambwe | 24.1 | 39.7 |  | Gwembe | 29.3 | 70.9 |
| Nyimba | 20.5 | 28 |  | Itezhi-tezhi | 18.2 | 26.9 |
| Petauke | 12.2 | 14.8 |  | Kalomo | 14.9 | 18.5 |
| Chiengi | 21.9 | 31.1 |  | Kazungula | 19.4 | 27 |
| Kawambwa | 19.7 | 27.2 |  | Livingstone | 13.4 | 16.5 |
| Mansa | 17.5 | 21.8 |  | Mazabuka | 9.6 | 12.2 |
| Milenge | 29.9 | 70.2 |  | Monze | 13.7 | 17.5 |
| Mwense | 18.4 | 24.2 |  | Namwala | 9.3 | 12.5 |
| Nchelenge | 15.9 | 19.9 |  | Siavonga | 22 | 30.8 |
| Samfya | 16.3 | 20.5 |  | Sinazongwe | 23 | 33.3 |
| Chongwe | 15.1 | 19.4 |  | Kalabo | 17.8 | 24.2 |
| Kafue | 8.4 | 10.3 |  | Kaoma | 13.9 | 17.8 |
| Luangwa | 24.7 | 41.7 |  | Lukulu | 20.1 | 28.8 |
| Lusaka | 3.8 | 4.5 |  | Mongu | 8.5 | 11.3 |
| Chama | 21.1 | 28 |  | Senanga | 19.3 | 28 |
| Chinsali | 18.4 | 23 |  | Sesheke | 16.2 | 21.7 |
| Isoka | 22 | 30.4 |  | Shang'ombo | 19.7 | 27.6 |
